# Lung Ultrasound in Pediatrics and Neonatology: An Update

**DOI:** 10.3390/healthcare9081015

**Published:** 2021-08-07

**Authors:** Angela Ammirabile, Danilo Buonsenso, Antonio Di Mauro

**Affiliations:** 1 Neonatology and Neonatal Intensive Care Unit, Department of Biomedical Science and Human Oncology, “Aldo Moro” University of Bari, 70100 Bari, Italy; 2 Department of Woman and Child Health and Public Health, Fondazione Policlinico Universitario A. Gemelli IRCCS, 00168 Rome, Italy; danilo.buonsenso@gmail.com; 3 Dipartimento di Scienze Biotecnologiche di Base, Cliniche Intensivologiche e Perioperatorie, Università Cattolica del Sacro Cuore, 00168 Rome, Italy; 4 Global Health Research Institute, Istituto di Igiene, Università Cattolica del Sacro Cuore, 00168 Rome, Italy; 5 Pediatric Primary Care, National Pediatric Health Care System, Via Conversa 12, 10135 Margherita di Savoia, Italy; dimauroantonio@msn.com

**Keywords:** lung ultrasound, pediatrics, ultrasonography, lung imaging, lung pathology, imaging

## Abstract

The potential role of ultrasound for the diagnosis of pulmonary diseases is a recent field of research, because, traditionally, lungs have been considered unsuitable for ultrasonography for the high presence of air and thoracic cage that prevent a clear evaluation of the organ. The peculiar anatomy of the pediatric chest favors the use of lung ultrasound (LUS) for the diagnosis of respiratory conditions through the interpretation of artefacts generated at the pleural surface, correlating them to disease-specific patterns. Recent studies demonstrate that LUS can be a valid alternative to chest X-rays for the diagnosis of pulmonary diseases, especially in children to avoid excessive exposure to ionizing radiations. This review focuses on the description of normal and abnormal findings during LUS of the most common pediatric pathologies. Current literature demonstrates usefulness of LUS that may become a fundamental tool for the whole spectrum of lung pathologies to guide both diagnostic and therapeutic decisions.

## 1. Introduction

The potential role of ultrasound (US) for the diagnosis of pulmonary diseases is a recent field of research. Traditionally, lungs have been considered unsuitable for ultrasonography for the high presence of air and thoracic cage that prevent a clear evaluation of the organ. This limitation can be overcome through the interpretation of artefacts generated at the pleural surface, correlating them to disease-specific patterns. For this reason, in addition to the wide use of the US for the assessment of different organs as a routine test, its utility for lung investigations is rapidly expanding, especially in the pediatric field. Currently, guidelines indicate chest X-rays (CXR) as first-line imaging modality for chest pathologies and computed tomography (CT) only in case of additional investigation, considering US useful only for the pleural effusion [1]. Moreover, lung ultrasound (LUS) represents a new subject in the point-of-care ultrasound (POCUS), particularly in emergency and critical-care medicine where it can be performed at bedside and interpreted in real time [2].

This review focuses on the description of normal and abnormal findings at LUS of the most common pediatric pathologies.

## 2. Principles of LUS

The peculiar anatomy of the pediatric chest favors the use of LUS for the diagnosis of respiratory conditions. The relatively unossified thorax of children, the large thymus gland, and the thinner subcutaneous tissue constitute acoustic windows for sonography: sternal and costochondral cartilages for anterior chest, mediastinum or intercostal scanning for lung and pleura, liver and spleen for the inferior thoracic cavity. Obviously, the process of ossification that normally occurs with age decreases the acoustic access to the thorax.

One of the main advantages of the expanded use of LUS in young children is the decreased exposure to radiation, and this allows test reproducibility without additional irradiation. Growing children are highly susceptible to DNA damages in rapidly dividing cells, and the risk of cancer development is much higher than in adults. Furthermore, LUS is a quick, portable, easy to operate, real-time imaging test that can be used either for diagnostic procedures or as a guide for interventional ones [3].

From the physical point of view, US is a high-frequency mechanical wave (over 20,000 Hz) generated by a transducer that successively receives the different echoes of human tissues. The interface represents the contact surface of two tissues with different acoustic impedance, i.e., tissue density multiplied by the velocity of wave propagation. Each interface reflects a specific amount of sound wave energy: higher differences in acoustic impedances result in greater energy reflection and echo signal (higher echogenicity). In human tissues, the high level of reflection that occurs in the presence of gas or bone prevents the definition of deeper structures (hyperechoic); at the chest–lung interface, 99% of energy waves are reflected in the presence of healthy lungs, allowing the visualization of pleural line [4]. A high-frequency linear probe of 10 MHz is used for LUS in neonates and young children to evaluate pleural line and subpleural space; a lower-frequency probe (1–5 MHz) can be further employed for diffuse or deeper diseases due to a wider and higher penetration [5]. Among the most important technical aspects, focus should be set at the level of interest, i.e., pleural line, and gain should be reduced to increase contrast for a better visualization of hyperechoic artifacts (A- and B-lines) or set to those used for parenchymatous organs in the presence of pneumonia [6]. Routinely, the 2D B-mode US (brightness modulation) is used to produce images composed by echoes of different brightness, but further tests can be performed with the color Doppler US for information about vascular flow or M-mode US (motion modulation) for data about diaphragmatic and pleural motion [7].

The examination is usually performed in the supine and lateral position according to the division of each hemithorax into three areas, further subdivided into upper and lower zones: anterior—between sternum and anterior axillary line, lateral—between anterior and posterior axillary line, and posterior—between posterior axillary line and spine. The dorsal assessment should always be performed, eventually in the prone or sitting position. It is important to systematically evaluate lungs through the main acoustic windows, i.e., suprasternal notch, parasternal regions, intercostal spaces, trans-diaphragmatic/sub-costal/sub-xyphoid scans, following a cranial–caudal orientation, or vice versa. Moreover, the sonographer should position the probe along different directions, mainly transverse (transducer along intercostal spaces) and longitudinal (transducer vertical to the ribs), and tilt it to avoid the ribs, allowing the screening of 70% of the lung parenchyma [8,9,10]. In addition to the aforementioned subdivision of hemithorax, other systems for lung division have been proposed to improve standardization (e.g., 12-zone or 14-zone system), especially in emergency settings. They take advantage of the use of scoring systems according to the quantification of B-lines in a specific area to establish disease severity [11,12].

## 3. Normal and Abnormal Findings at LUS

The sonographic examination of the lung should always be clinically driven to perform a focused evaluation and reach a higher accuracy in the diagnosis of specific pulmonary diseases; the knowledge of normal artefacts produced by LUS by a healthy lung is fundamental to distinguish them from pathologic patterns [13].

The first step is the identification of the pleural line, usually well visualized because of its superficial localization above the aerated lung. It appears as a horizontal hyperechoic line between two adjacent ribs that shows a posterior shadowing: this is called bat sign, visible in the B-mode US, and it assures the correct positioning of the probe on the longitudinal axis. If the probe is positioned in an intercostal space, the pleural line appears without interruptions. Pleural visualization in the M-mode US creates another physiological sign, defined as lung sliding, seen as a bright line. It is due to the movement of the parietal pleura against the visceral one during respiration (to-and-from movement) along the cranio-caudal axis. In addition, the seashore sign shows the presence of lung sliding: the superficial motionless area above the pleura generates horizontal lines—waves, and the mobile area under the pleura appears granular for the reflection of the movement—sand. A-lines represent the other important normal artefact generated by the large difference in acoustic impedance at the interface between pleura and lung. They are hyperechoic horizontal lines deeper than pleural lines, parallel and equidistant from one another that are able to exclude the presence of lung pathologies in the scanned area.

**Figure 1:**
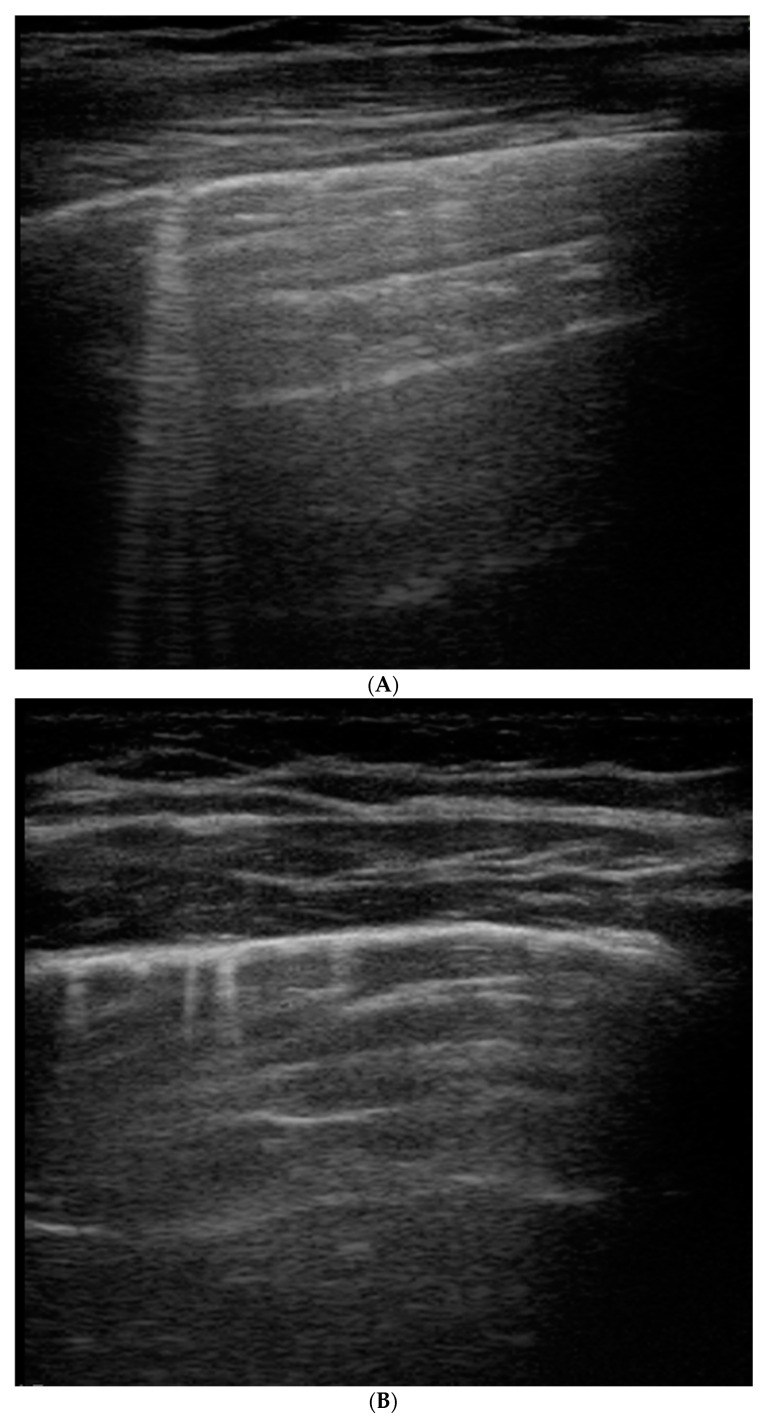

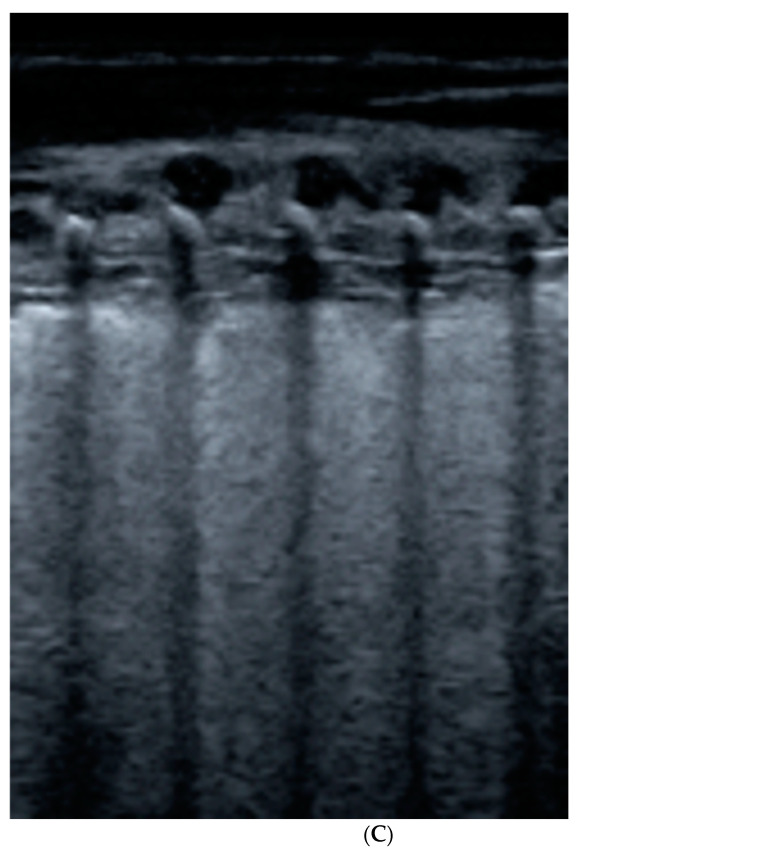
B-lines: vertical hyperechoic artifacts, extending from the pleura that erase normal A-lines (horizontal hyperechoic artifacts). They are the sign of increased interstitial fluid content and thickening of interlobular septae. (**A**) Deep B-lines, (**B**) short B-lines, (**C**) compact B-lines: the number of these artifacts is proportional to the decrease in air content with a tendency to confluence in severe alveolar–interstitial syndrome.

The in-depth description of abnormal findings at LUS can help the recognition of specific lung pathologies. First, B-lines are commonly found in respiratory disease as signs of increased lung interstitial fluid content and thickening of interlobular septae or scarring of the septae in chronic diseases: their number is proportional to the decrease in air content. These comet-tail artefacts are laser-like, hyperechoic, vertical lines that project from the pleural line and completely remove the normal A-lines. They move simultaneously with pleura during respiration, and their unilateral or bilateral distribution is suggestive of specific pathologies (unilateral: pneumonia, bilateral: edema, transient tachypnea of the newborn—TTN). The presence of fewer than three B-lines has no pathological meaning, especially in the first 48 h of life for the high presence of fluid in the lungs of neonates. Moreover, the appearance of compact B-lines indicates a severe alveolar–interstitial syndrome, also known as white lung, up to the complete lung consolidation for the absence of air in which the lung parenchyma becomes an acoustic window for its direct visualization. The US appearance of this latter pulmonary anomaly is defined as lung hepatization, so called for the similar appearance of the abnormal lung parenchyma to the normal hepatic one: visualization of a hypoechoic region with blurred margins and wedge-shaped borders (Figure 1). In this case, air bronchograms appear as hyperechoic structures for the presence of air in bronchioles, and their features vary according to the specific etiology of lung consolidation. The alveolar consolidation should not be deep, but it must reach the pleural surface to allow visualization through the intercostal window. Another pathological finding at LUS is the lung pulse, i.e., the synchronized movement of the pleura with the cardiac rhythm at M-mode US for the transmission of cardiac activity on lung parenchyma. It is not present in a normally aerated lung for the dominant action of lung sliding over the cardiac vibration, and if present, it is a sign of absence of ventilation [14]. Summarizing, a black appearance means healthy lung, a mixed black and white appearance is associated to a moderate disease, and a completely white lung is a marker of severe respiratory distress.

## 4. Application of LUS in Pediatric Diseases

### 4.1. Pleural Effusions

**Figure 2:**
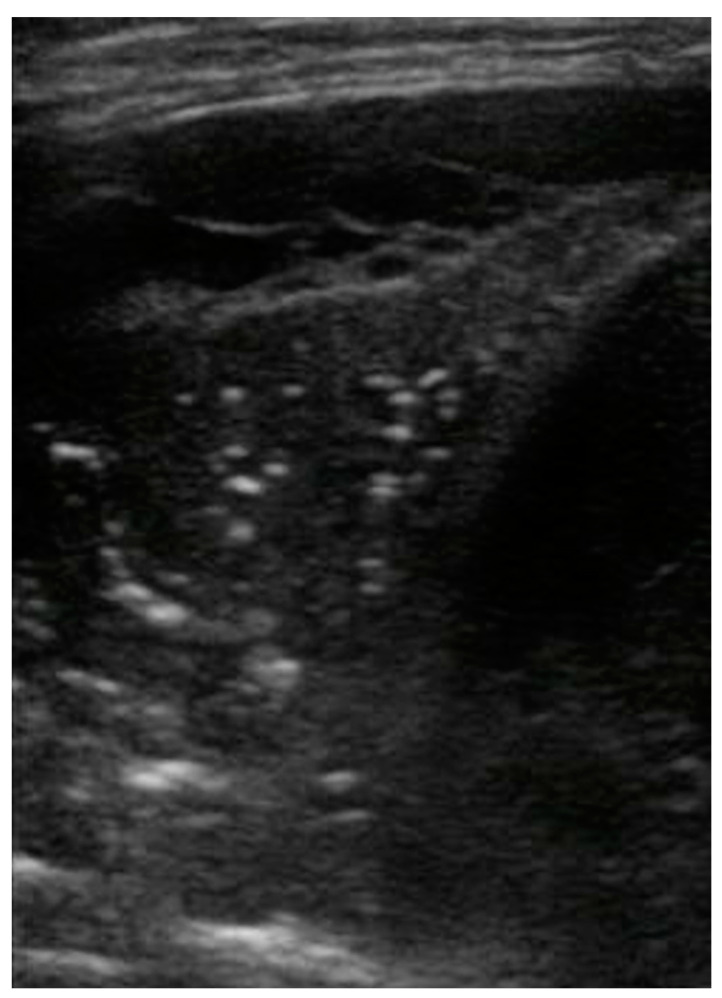
Complex pleural effusion associated with bacterial pneumonia: presence of fibrinous strands and septations (echogenic appearance) in the typical anechoic image related to fluid.

The utility of LUS for the evaluation of pleural effusions has been already established [15]. The pleural space is well visualized because of its superficial location to the lung parenchyma. Its high sensitivity allows the detection of small amounts of effusions around 3–5 mL, and it is more sensitive than CXR, considering the challenge of differential diagnosis of an opacified hemithorax and a minimal presence of 150 mL of fluids [16]. The sonographic appearance of pleural effusion varies according to the composition of the fluid, also allowing a classification of the disease. A simple pleural effusion is shown as an anechoic image for the exclusive presence of transudate fluid without any kind of debris, and it can change shape according to breathing or changes in position. Complex effusions are characterized by the presence of exudate and are further divided into low-grade for the presence of mild echogenic floating particles for infections or hemorrhage or high-grade for complex echogenic content related to organized infections or empyema. The latter type can show septations, fibrinous strands, loculation, and eventually pleural thickening in the so-called malignant effusions for pleural metastasis (Figure 2) [17,18]. Considering their incidence in children, LUS can have a role in the imaging of parapneumonic effusion and empyema with implications on treatment [19,20]. However, LUS cannot predict the presence of blood, chyle, or protein in the fluid, even if a US-guided thoracentesis can be performed in a safe and easy way [21]. Color Doppler could be used to distinguish an echogenic fluid from a solid collection: a fluid-color sign is positive only in case of mobile debris. In addition, the absence of the mirror image artifact can support in the diagnosis of pleural effusion: liver parenchyma is normally reflected on the posterior costophrenic angle through the diaphragm (strong reflector) when there is a normally aerated lung parenchyma [22].

### 4.2. Pneumothorax

**Figure 3:**
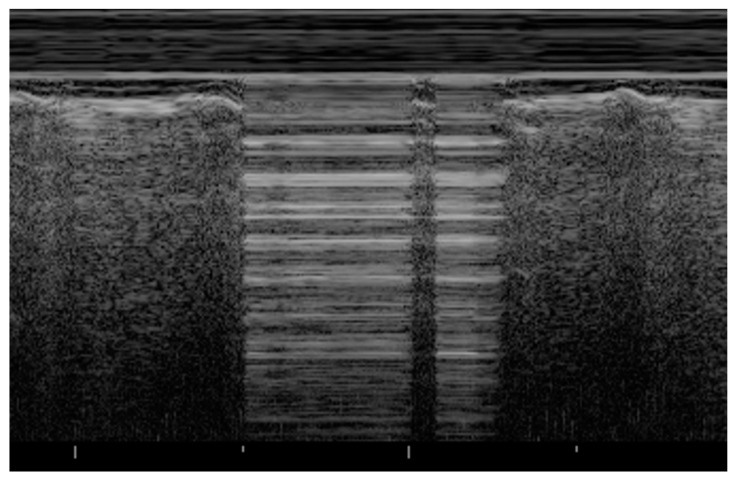
Pneumothorax: evidence of lung point, i.e., the transition point from the typical LUS pattern of PNX to the normal one.

Pneumothorax can be easily studied with LUS with a higher accuracy than supine anteroposterior CXR [11,23]. Specifically, LUS performances are better in neonates than adults, reaching a sensitivity of 96.7% (vs. 82.9%) and a specificity of 100% (vs. 98.2%) [24]. The main pathological finding is the absence of lung sliding on B-mode imaging for the presence of air between the two pleural layers that inhibit the to-and-from movement and the related underlying granular pattern. The consequence is the absence of a dynamic image, substituted by a static posterior acoustic shadowing called barcode or stratosphere sign for the horizontal lines. This pattern has a negative predictive value of 100% for pneumothorax, and its absence is sufficient to rule out the diagnosis [25]. Lung point is another sign needed to confirm the diagnosis with a 100% specificity, visible as the transition point from the typical LUS pattern of pneumothorax to the normal one that occurs where the lung adheres again to the parietal pleura [26]. In their cohort of 49 neonates with respiratory distress, Cattarossi et al. described a correlation between the location of lung point and the pneumothorax extension, using the mid-axillary line as cut-off: a transition point at or beyond that line is an expression of a large pneumothorax [23]. According to international evidence-based recommendations, the absence of B-lines and lung pulse are included in the lists of additional sonographic signs (Figure 3) [11].

- Seashore sign (lateral): echogenic pleural line divides the image in the motionless part represented by horizontal lines (sea waves) and the part below that appear granular (sand) as the normal to-and-from motion of the lung is reflected over that area (normal lung sliding).
- Barcode sign (central): absence of a dynamic image, substituted by a static posterior acoustic shadowing (horizontal lines).

### 4.3. Pneumonia

Pneumonia can be considered a fatal disease during the neonatal period, accounting for 10% of global child mortality [27], and LUS could be a useful tool in its evaluation [9,28,29]. Pathogens may be acquired in different ways, such as intrauterine or postpartum, and the non-specific signs of neonatal pneumonia increase the difficulty of diagnosis, especially in the presence of respiratory distress for distinction with respiratory distress syndrome (RDS) and TTN [30]. General features are epithelial injury, fluid leakage, and alveolar–interstitial edema that are visualized in a LUS pattern characterized by multiple B-lines and hypoechoic, liver-like consolidation with irregular margins (shred sign), disappearance of A-lines. Generally, the pleural line is not visible in the affected area, lung sliding is absent, and a concomitant pleural effusion is present in one third of cases. Moreover, a typical feature of pneumonia is the presence of dynamic air bronchograms, i.e., branching linear echogenicities that move with respiration, as signs of air trapping in patent bronchi, differently from atelectasis. If fluid or mucous material accumulates within bronchi as in necrotizing or post-obstructive pneumonia, the sonographic fluid bronchogram appears, showing hypoechoic branching structures.

**Figure 4:**
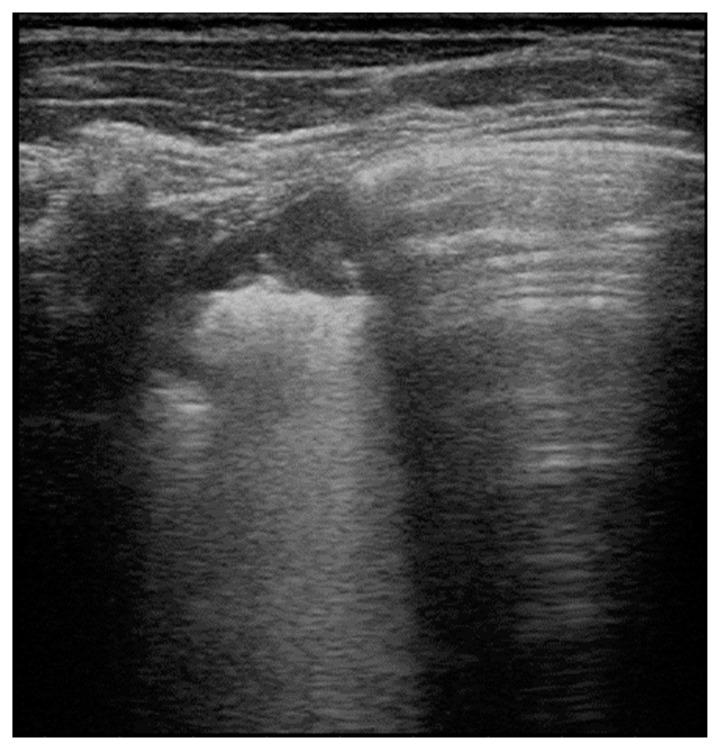
Mycoplasma pneumonia: area of consolidation (lung hepatization) with blurred margins and disappearance of pleural line. Adjacent to the affected area, evidence of normal A-lines, i.e., hyperechoic horizontal lines deeper than visible pleural line, parallel and equidistant from one another that are able to exclude the presence of lung pathologies in the scanned area.

Different meta-analyses have demonstrated that LUS can reach a sensitivity of 95.5–96.7% and a specificity of 87.3–95.3% in the pneumonia diagnosis and up to 100% sensitivity and 94% specificity in the follow-up evaluation [28,31,32]. Additional advantages of LUS regard the high interobserver concordance, also with novice sonographers due to rapid learning curves [33,34]. According to Liu et al., the presence of a large area of lung consolidation with irregular margins can diagnose severe neonatal pneumonia with 100% sensitivity and 100% specificity [35]. Considering that CXR is used for diagnosis confirmation, common endpoints of multiple studies were the evaluation of the overall agreement between CXR and LUS and the comparison of the diagnostic performances. Some studies, initially conceived as non-inferiority trials, proved that LUS had a higher reliability in terms of pneumonia diagnosis [36,37,38,39,40], eventually identifying false-negative patients at CXR that needed a CT scan for diagnosis confirmation [41]. In particular, LUS demonstrated a higher sensitivity than CXR, consistent with data reported in literature about a major rate of false-negative and interobserver variability [42,43,44]. The estimated concordance between these two imaging techniques reached a Kappa Cohen coefficient = 0.89 [45]; interestingly, this value is similar to those found by Bloise et al. for the right lung (K = 0.88), but not for the left lung (K = 0.70), probably due to the interference of the heart shadow [46]. However, a possible limitation of LUS regards a lower sensitivity for the detection of lesions that do not reach the pleural surface, mainly as perihilar pneumonia for its deep localization, usually not visible for the presence of air (Figure 4) [36,45]. LUS can be a better diagnostic method for the assessment of pneumonia complications, also for an earlier detection than CXR [17]: necrotic areas appear as hypoechoic areas without recognizable Doppler flow [47] and lung abscesses as a thick-wall structure filled with anechoic fluid. Large abscess can take advantage of LUS both for diagnosis and treatment with US-guided aspiration and drainage [48]. The performances of LUS have been compared to those of CT, which is usually employed in the evaluation of complicated pneumonia to assess parenchymal and pleural abnormalities [49]. LUS appeared to be positively correlated with CT in complicated cases, up to full agreement (K = 1), estimating the degree of impaired perfusion as sign of lung necrosis (AUC = 0.98, r = 0.704) or the presence of fibrin strands within a parapneumonic effusion as expression of empyema [47,50,51].

Even if imaging techniques cannot identify the etiologic factors, LUS can be suggestive of pneumonia etiology, especially if integrated with clinical data; despite some overlapping features, [29,52] LUS has a higher sensitivity for bacterial pneumonia than viral ones (91% vs. 78.4%, respectively) [53]. The main sonographic features of bacterial etiology are lung consolidations (>0.5 cm), air bronchogram, and shred sign, i.e., signs of an alveolar pathology; pleural effusion is often associated. On the other hand, interstitial disease is suggestive of a viral etiology, represented by pleural abnormalities (irregularity or thickening), variable number, and coalescence of B-lines and multiple small subpleural consolidations (<0.5 cm), frequently bilateral. B-lines are the sonographic sign of thickened interlobular septae that appear as ground–glass opacities on a CT scan [54]. The same interstitial pattern has been described in coronavirus disease 2019 (COVID-19), with a prevalence of lung abnormalities in the posterior regions and at lung bases that are easily evaluated with LUS [55]. Despite the paucity of studies about this new infection in children (characterized by lower incidence and severity [56]), it appears that sonographic features of pediatric COVID-19 are non-specific and similar to those described in adults: B-lines with a tendency to coalescence; subpleural consolidations associated with pleural line irregularities [57,58,59,60,61]. Moreover, LUS was found to have a higher sensitivity than CXR in the diagnosis of COVID-19 (83.33–88.9% vs. 25–51.9%, respectively), improving early detection [52,62,63].

### 4.4. Atelectasis

**Figure 5:**
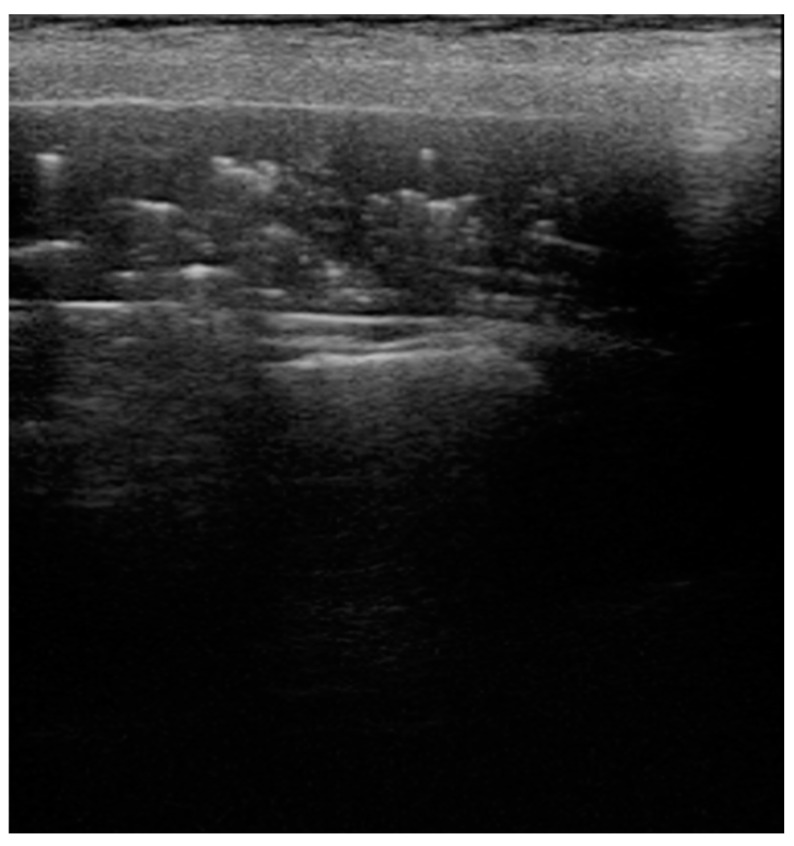
Atelectasis in a patient with neuromuscular disease: presence of static horizontal bronchograms that do not move with respiration.

The term atelectasis describes the collapse of lung parenchyma as the cause of impairment of gas exchange and loss of lung volume. The most common mechanism in infants is airway obstruction for accumulation of mucus plug or meconium, causing air trapping in the distal airways and pulmonary collapse after air resorption from blood. Resorptive atelectasis is strictly associated with mechanical ventilation for insufficient pressures. It cannot be distinguished from consolidation using only US due to a similar pattern of low echogenicity and hepatization, variable degree of B-lines, absence of lung sliding, and pleural line anomalies. A peculiar feature of atelectasis is static bronchograms that do not move with respiration, seen as branching structures in the context of subpleural consolidations of variable sizes (Figure 5). Lung pulse can be considered among the most important signs of atelectasis that can reach a specificity of 100% for the diagnosis of complete atelectasis [64,65]. LUS has shown to correlate with magnetic resonance imaging (MRI) for the diagnosis of anesthesia-induced atelectasis in children (K = 0.75), reaching an accuracy of 88% [66].

### 4.5. Congenital Lung Malformations

Congenital pulmonary anomalies represent a group of lesions due to a variable degree of airway obstruction in utero and subsequent dysplastic changes [67]. They can cause severe respiratory distress and admission to the neonatal intensive care unit (ICU) [68]. Congenital lung malformations (CLM) can be prenatally diagnosed with US screening, eventually complemented with antenatal MRI for a more accurate evaluation [69]. They are usually discovered during the mid-second-trimester US (19–22 weeks of pregnancy) with well-defined sonographic patterns to assess location, size, composition (solid, cystic or mixed), and blood supply. Related complications can also be evaluated, mainly pleural effusion, signs of fetal hydrops, and mediastinal shift, and, if severe, they can be an indication for prenatal intervention [70,71,72]. However, a postnatal confirmation is always required with CXR or CT, even in asymptomatic newborns, to choose the appropriate type of management [73]. To date, few studies have applied LUS in the evaluation of CLM, demonstrating a good correlation with CT findings [68,74].

Pulmonary sequestrations are abnormal lung segments without connection to the tracheobronchial tree but connected with the systemic arterial supply. The intra-lobar type is contained in the native pleural lining, usually located in the lower lobes and towards the left side, while the extra-lobar type is contained in a separate pleural lining and could also be located under the diaphragm. The latter type is more common in infants. They are discovered for a high tendency of infection or for the presence of pulmonary opacities at CXR. The color Doppler is particularly useful for the diagnosis, because the demonstration of connection with the systemic arterial supply is mandatory, usually from the descending aorta. The sonographic appearance is variable from hypoechoic, solid masses, to anechoic, cystic formations.

**Figure 6:**
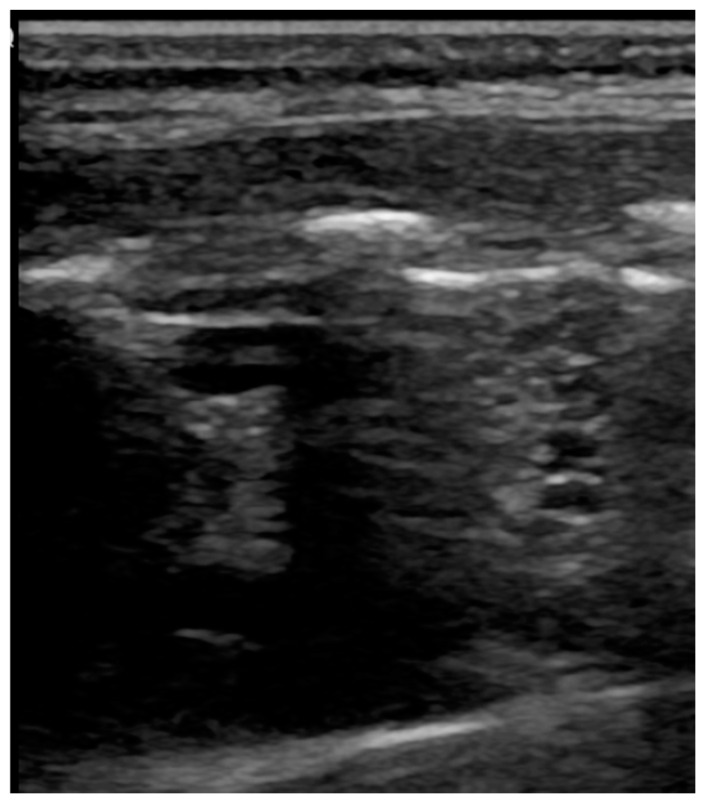
Congenital pulmonary airway malformations: multiple cystic lesions, not found in other pulmonary pathologies.

Congenital pulmonary airway malformations are cystic masses of lung parenchyma filled by proliferating an immature bronchial structure that could interfere with normal lung development due to a strong mass effect. The specific sonographic finding is the presence of single or multi-loculated cystic lesions, not found in other pulmonary conditions, eventually associated with consolidations (Figure 6) [68,74].

### 4.6. Congenital Diaphragmatic Hernia

Similarly to congenital pulmonary anomalies, the diagnosis of congenital diaphragmatic hernia (CDH) generally occurs by fetal US with a detection rate of 60% [75], and an early postnatal LUS can be useful in case of missed diagnosis. After birth, point-of-care ultrasound supports a differential diagnosis for respiratory symptoms [76,77]. Two types of CDH have been described according to the position of the abnormality: anteromedial—Morgagni, or posterolateral—Bockdalek. The defect is demonstrated by an interruption of the hyperechoic diaphragmatic line that prevents the normal lung sliding and erases A-lines in the affected area. Sometimes, abdominal organs, such as liver and spleen, can be recognized in the thorax. In CDH evaluation, subcostal scans have to be performed in addition to the trans-thoracic view [78].

### 4.7. Neonatal Pathologies

The application of LUS for the evaluation of neonatal pathologies is becoming more and more important, considering it as a possible diagnostic challenge for the poor sensitivity and specificity of signs and symptoms [79].

An important clinical application is the study of RDS, one of the main causes of neonatal mortality, as described in the European Consensus Guidelines [80]. It occurs especially in preterm infants for deficiency of surfactants and lung immaturity, leading to a decreased gas exchange and alveolar collapse. The main sonographic features are widespread compact B-lines and an associated white lung appearance, a thickened and irregular pleural line, and multiple subpleural lung consolidation are seen as small hypoechoic areas. These patterns do not improve immediately after the administration of surfactants [81]. Additional findings of severe cases of RDS (grade 3 and 4) are widespread, with deep consolidations and presence of lung pulse. A recent study demonstrates that a LUS score calculated in the first hours of life can predict the need for intubation and respiratory support (significant correlation with oxygenation indices) or for surfactants (AUC 0.93–0.97 for preterm babies less than 34–35 weeks), considering the abundance of B-lines as the main indicator of severity [82,83].

TTN has been extensively evaluated for LUS imaging [84,85,86]. This disease, also called wet lung, is the cause of postnatal respiratory distress due to a delayed clearance of fetal lung liquid, and the main risk factors are elective caesarean section and prematurity for decreased activity of the lung epithelial sodium channel responsible for fluid resorption (ENaC). The general presentation is mild or transient respiratory distress that can persist up to a few days. The pathognomonic finding at LUS is the double lung point as a sharp demarcation of changes in echogenicity, resulting from highly compact B-lines in the inferior part and rare B-lines in the upper part. It can reach a sensitivity and a specificity of 100% and facilitate the differential diagnosis with RDS, together with the presence of a regular pleural line in TTN [87].

Meconium aspiration syndrome (MAS) is another condition that occurs especially in term or post-term infants. In fact, after inhalation of meconium from the amniotic fluid, the fetus can suffer from hypoxia, acidemia, and infection, secondary to airway obstruction or surfactant dysfunction. MAS manifestations at LUS are similar to those of pneumonia, such as irregular subpleural consolidations, compact B-lines, air bronchograms, even if they vary according to the severity. There are generally dynamic signs without a fixed distribution pattern, probably due to meconium movement inside the lungs, and with good correlation with X-rays findings [88].

Another well-studied neonatal disease is cronchopulmonary dysplasia (BPD), i.e., a chronic lung disease characterized by bilateral changes in lungs, particularly common in preterm delivery that need oxygen support for more than 28 days [89]. It occurs due to a block of pulmonary development that predisposes affected children to infections, oxidant injury, and poor nutrition. BPD can also be considered a common complication of RDS. The specific LUS pattern is based on inhomogeneous subpleural consolidations and fragment-like echoes, irregular pleural line, simultaneous presence of areas with normal parenchyma or compact B-lines, or white lung [90]. Considering that BPD occurs especially in the peripheral areas, a transabdominal approach is useful for the visualization of lung bases. Multiple studies estimated the predicted values of serial LUS in preterm babies born before 32 weeks through multiple sonographic examinations in the first 28 days of life, according to the BPD definition. They concluded that monitoring of lung aeration and function through LUS scores in the first two weeks of life, mainly at 7 and 14 days (AUC 7 days 0.74–0.97; AUC 14 days 0.73–0.95), significantly predicts the development of BPD and is useful to stratify high-risk newborns [91,92,93,94].

Few studies have investigated the application of LUS in the diagnosis of pulmonary hemorrhage of the newborn (PHN), a potentially life-threatening condition that usually occurs in the first week of life [95,96]. One of the major sonographic features is the shred sign due to a blurred margin between aerated and consolidated parenchyma: based on the results of Ren et al., it occurs in more than 90% of affected children and exhibited a specificity of 100% and a sensitivity of 91.2% in PHN diagnosis. Pleural line abnormalities and A-lines disappearance are present, possibly associated with bloody pleural effusions, pulmonary atelectasis, and alveolar–interstitial syndrome [97].

### 4.8. Bronchiolitis

Bronchiolitis is a viral disease that affects the lower respiratory tract during the first year of life with a peak incidence between 3 and 6 months of age, and it is considered the first cause of children hospitalization. The most common cause is the respiratory syncytial virus, a single-stranded RNA virus [98]. According to the most recent guidelines, the diagnosis of bronchiolitis is based only on anamnesis and clinical evaluation [99]. The additional use of imaging methods is restricted to severe cases, such as pulmonary complications or before the admission to ICU: CXR is commonly performed. Apart from the exposition to radiations, Schuh et al. reported multiple limitations of CXR in the evaluation of bronchiolitis, including increased subsequent antibiotics administration for a similar appearance of consolidation and atelectasis [100]. LUS has not been included in the diagnostic algorithm yet, even if the current literature is focused on the evaluation of the possible benefits derived from its routine use. In fact, considering a diagnostic value equal or superior to CXR, LUS can be the first choice if imaging tests are required [29,101].

**Figure 7:**
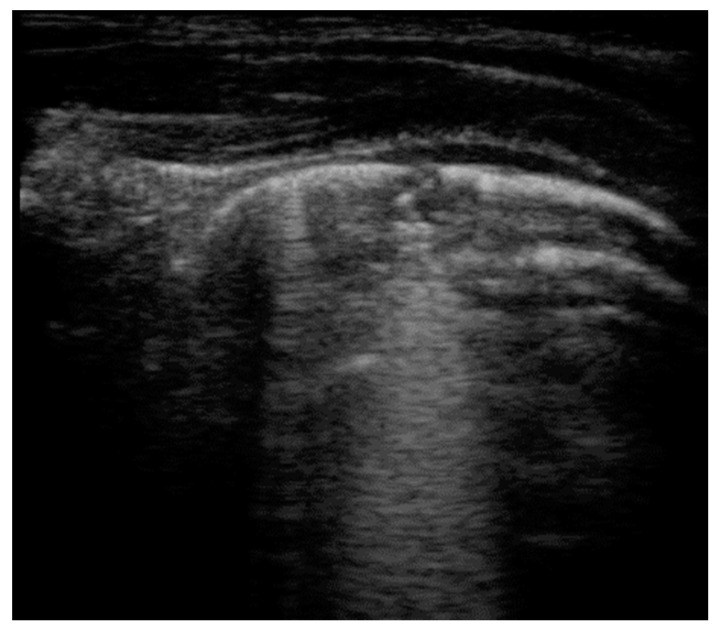
Bronchiolitis: small subpleural consolidation in a newborn, expression of a disease moderate in severity.

Caiulo et al. performed the first study about the sonographic pattern of bronchiolitis, demonstrating a good correlation with clinical findings and higher performances than CXR, especially in complicated cases, for the detection of small pleural effusions and pneumothorax. The typical LUS pattern of bronchiolitis varies according to the disease severity: a progressive increase in B-lines number with a tendency to coalescence into single subpleural consolidation (small ones < 1 cm or large ones > 1 cm with tissue-like echo-structure and ultrasonographic bronchograms) and areas of white lung in the moderate disease or multiple lung consolidations in severe forms, eventually associated with pleural line anomalies (thickening and irregularity) and pleural effusions (Figure 7). LUS can be also useful in the monitoring of treatment efficacy because the disappearance of abnormal sonographic findings is correlated with clinical improvement [5,102]. The abnormalities are usually found in the inferior and posterior areas of the lungs, probably explained by gravity or the obligate supine position of the child. However, CXR can still have some advantages in the evaluation of hilar areas and lung hyperinflation [103].

Starting from the knowledge of most common sonographic findings, ultrasound severity scores can be developed to correctly stratify affected children and to predict the clinical course of bronchiolitis; they can also be compared with clinical scores based on the most common symptoms and signs with a reported agreement of 90.6%. LUS allows for the identification of patients in need of supplemental oxygen with high sensitivity and specificity (96.6% and 98.7%, respectively) and correlates with the length of hospital stay [104,105,106,107,108]. According to Bueno-Campana, the identification of at least one consolidation >1 cm in the posterior areas has a relative risk of 4.4 for the need of non-invasive ventilation [109]. The sonographic evaluation of bronchiolitis could benefit from additional diaphragmatic scans to evaluate some parameters that appear to be related to outcome, such as diaphragmatic thickening, excursion, and velocity of contraction. In fact, abnormal values indicate diaphragmatic dysfunction due to increased respiratory load and possible progression toward respiratory failure [110].

Moreover, LUS can diagnose secondary pneumonia as a complication of bronchiolitis to justify antibiotics administration, reaching an AUC of 0.92 and a sensitivity and a negative predictive value of 100%. If only consolidations > 1 cm are taken into consideration, the specificity of LUS appeared to be higher than that of CXR (98.4% vs. 87.1%, respectively) with a decrease in sensitivity and negative predictive value (80% vs. 92.4%, respectively) [111].

## 5. Discussion

The instrumental evaluation of pulmonary pathologies has been always performed with CXR, exposing patients to radiation. In fact, the use of LUS has started to be considered as an alternative imaging test for a few years, overcoming the traditional view of the lung as an unsuitable organ for sonographic examination due to its elevated air content. The first applications of LUS have been described in the diagnosis of pneumothorax, pleural effusion, and lung consolidation as defined in the “International evidence-based recommendations for point-of-care lung ultrasound”, published in 2012. This report is based on deep literature research over the last 20 years with the aim of providing clinical indications for the use of LUS, considering its main advantages of safety and quickness [11]. The first attempt in the use of pulmonary sonography dates back to 1990, defined as “a new method of diagnosing hyaline membrane disease in newborns” [112]. After this trial, studies about LUS applications have been conducted previously in adults [25] and more recently in children [87,113].

Over the years, POCUS has been gaining popularity in the pediatric clinical practice, as demonstrated by the development of guidelines for its use in critically ill children [114]. These recommendations emphasize the current idea of the double role of this imaging tool. On one hand, the classical descriptive LUS applications diagnose neonatal pulmonary pathologies in a qualitative way, as an alternative to traditional radiographic procedures. On the other hand, the new functional LUS applications, also known as semi-quantitative ones, allow us to predict disease evolution through sonographic scores (e.g., monitoring of pulmonary edema [115] or prediction of ICU length [116] after cardiac surgery), to guide therapeutic decisions (e.g., surfactants need in RDS [117]), or to perform invasive procedures (e.g., thoracentesis or chest tube positioning) [118].

From the beginning of 2020, LUS has demonstrated additional benefits in the first-line evaluation of suspected COVID-19 patients, either adults and children. In fact, it is a bedside evaluation test that can also be performed in critically ill patients without further movements inside the hospital, increasing the safety of healthcare workers [52,59,119].

As previously reported, studies have reported a rapid learning curve for novice sonographers that can perform accurate scans after a few hours of teaching and practice [33,120]. These advantages can be summed up to the affordability and the portability of ultrasonography that make its use feasible in low- and middle-income countries. In particular, lung ultrasonography can have a central role in the diagnosis and management of pneumonia in resource-limited settings to reduce morbidity and mortality in children [121].

LUS should be included in the diagnostic algorithms of pulmonary pathologies to decrease radiation exposure, knowing that it cannot substitute completely radiographic tests [122]. An intrinsic limitation of pulmonary sonography concerns diseases of the hilum or central airways, because these areas are far from the pleura and are not visualized through trans-thoracic scans. Moreover, ultrasound is an operator-dependent technique, and it requires a targeted training of sonographers that can have a significant impact on LUS diagnostic accuracy [123]: for this reason, a common aim in studies about LUS use is inter-observer agreement [124]. Then, a recent study suggests that healthy children can show mild abnormalities at LUS in the first 6 month of age, i.e., presence of vertical B-lines in the absence of underlying disease for lung immaturity. This aspect can lead to misdiagnosis of diseases, especially in inexperienced sonographers [125]. Further studies are needed to establish a definitive role of sonography in the different aspects of pulmonary diseases, from diagnosis to therapeutic interventions.

## 6. Conclusions

The knowledge about LUS usefulness is becoming wider, and it would represent a fundamental tool for the whole spectrum of lung pathologies to guide both diagnostic and therapeutic decisions. In particular, its application in the pediatric field is well studied to avoid unnecessary child exposure to ionizing radiation for its associated increased risk in cancer development.

## Data Availability

Not applicable.
